# Environmentally friendly method to grow wide-bandgap semiconductor aluminum nitride crystals: Elementary source vapor phase epitaxy

**DOI:** 10.1038/srep17405

**Published:** 2015-11-30

**Authors:** PeiTsen Wu, Mitsuru Funato, Yoichi Kawakami

**Affiliations:** 1 Department of Electronic Science and Engineering, Kyoto University, Kyoto 615-8510, Japan

## Abstract

Aluminum nitride (AlN) has attracted increasing interest as an optoelectronic material in the deep ultraviolet spectral range due to its wide bandgap of 6.0 eV (207 nm wavelength) at room temperature. Because AlN bulk single crystals are ideal device substrates for such applications, the crystal growth of bulky AlN has been extensively studied. Two growth methods seem especially promising: hydride vapor phase epitaxy (HVPE) and sublimation. However, the former requires hazardous gases such as hydrochloric acid and ammonia, while the latter needs extremely high growth temperatures around 2000 °C. Herein we propose a novel vapor-phase-epitaxy-based growth method for AlN that does not use toxic materials; the source precursors are elementary aluminum and nitrogen gas. To prepare our AlN, we constructed a new growth apparatus, which realizes growth of AlN single crystals at a rate of ~18 μm/h at 1550 °C using argon as the source transfer via the simple reaction Al + 1/2N_2_ → AlN. This growth rate is comparable to that by HVPE, and the growth temperature is much lower than that in sublimation. Thus, this study opens up a novel route to achieve environmentally friendly growth of AlN.

Due to its high thermal conductivity, excellent dielectric properties, and high chemical stability, aluminum nitride (AlN) is often used as electronics packages and heat sinks. In addition, AlN has recently been considered as an optoelectronic material due to its direct wide bandgap of 6.0 eV at room temperature and piezoelectricity. In an effort to develop energy saving devices, AlN-based deep-ultraviolet light-emitting devices^1^,^2^,^3^,^4^,^5^,^6^,^7^,^8^,^9^,^10^,^11^,^12^,^13^, high electron mobility transistors^14^,^15^, sensors^16^, etc. have already been exploited.

Despite extensive studies, AlN-based device performance is currently limited; for example, the external quantum efficiency (EQE) of AlGaN light-emitting diodes (LEDs) is at most ~15% in the spectral range of 260–300 nm and decreases at shorter wavelengths. (It is noteworthy that EQE of InGaN blue LEDs has exceeded 80%^17^). Because AlN substrates are immature and quite expensive, foreign substrates such as sapphire and silicon carbide (SiC) are widely used in devices^1^,^2^,^3^,^4^,^5^,^6^,^7^,^10^,^11^,^14^,^15^. Unfortunately, the lattice and crystallographic mismatches between AlN and a foreign substrate induce threading dislocations, which are on the order of 10^9^–10^11^ /cm^2^ in the epilayer. One effective way to improve device performance is to further develop AlN bulk single crystals as the substrate, which may significantly reduce the number of threading dislocations in the device.

Currently, there are two main AlN bulk crystal growth techniques: hydride vapor phase epitaxy (HVPE)^18^,^19^,^20^,^21^ and sublimation^22^,^23^,^24^,^25^,^26^,^27^. Although these method have progressed, they still have limitations. Because the reaction of Al + 1/2N_2_ → AlN is seemingly inactive, especially due to the inert nature of N_2_ gas, AlN crystal growth usually involves processes to activate Al and N. For example, HVPE uses NH_3_ and Al-trichloride generated via a reaction between Al and HCl, while sublimation requires AlN powders and extremely high growth temperatures around 2000 °C. [This is also true for other thin film growth techniques. Typically, molecular beam epitaxy (MBE) adopts a plasma to excite N_2_^28^, whereas metalorganic vapor phase epitaxy (MOVPE) uses Al-metalorganic and NH_3_^29^,^30^]. That is, although AlN itself is an ecological material, the present growth processes are environmentally burdensome (e.g., employing toxic gases and high temperatures).

In addition to growth methods, how to nucleate AlN is another critical issue. Similar to thin film growth, homoepitaxial and heteroepitaxial nucleations are possible for bulk AlN single crystals. For HVPE, homoepitaxy on AlN substrates grown by sublimation^8^,^20^ and heteroepitaxy on sapphire or SiC substrates^18^,^19^,^21^ have been investigated. For sublimation, spontaneous nucleation of AlN and subsequent homoepitaxy^22^,^23^,^24^, and heteroepitaxial nucleation on SiC^25^,^26^,^27^ have been demonstrated. Homoepitaxial nucleation realizes a much higher crystalline quality with a dislocation density of 10^3^–10^5^ /cm^2^, but enlarging AlN crystals to a size suitable for industrial applications remains a challenge. On the other hand, heteroepitaxial nucleation may involve more dislocations, but can realize a larger AlN diameter because more than three-inch sapphire and SiC are commercially available.

Herein we propose a novel route to realize environmentally friendly growth of AlN using only elementary Al and N_2_ gas as the source precursors. Initially, thermodynamic analysis is used to examine the reactivity between Al and N_2_ to create AlN. Then a growth apparatus is constructed to realize the reaction Al + 1/2N_2_ → AlN. Using the developed apparatus, AlN single crystals are successfully grown at a rate of ~18 μm/h at 1550 °C. The growth rate is comparable to that by HVPE, and furthermore, the growth temperature is much lower than that in sublimation (~2000 °C). We employed heteroepitaxy on sapphire substrates as a nucleation method because this study demonstrates proof-of-concept AlN growth and heteroepitaxy is easier and more cost effective than homoepitaxy for this purpose.

## Results and Discussion

### Concept and construction of growth apparatus

**Figure 1:**
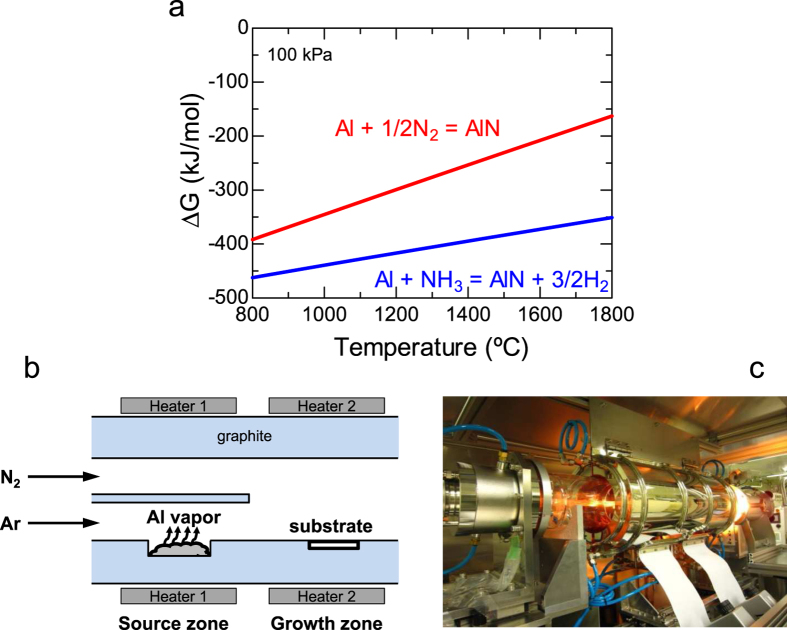
Fundamental idea of the newly developed AlN crystal growth apparatus. (**a**) Calculated Δ*G* of the reaction Al + 1/2N_2_ → AlN and for comparison, the reaction Al + NH_3_ → AlN + 3/2 H_2_. Negative Δ*G* values indicate that both are spontaneous. (**b**) Schematic of the growth apparatus design in which direct nitridation of the Al source is avoided as Ar gas transfers Al vapor to the growth zone. (**c**) Photograph of the growth apparatus in operation.

A simple thermodynamic calculation on the standard Gibbs free energy change (Δ*G*) suggests that the reaction Al + 1/2N_2_ → AlN may occur at a reasonable temperature; Fig. 1a shows the calculated result at 100 kPa as well as the Δ*G* for the more common reaction Al + NH_3_ → AlN + 3/2H_2_. (The enthalpy and entropy values for the calculations are from ref. 31.) Because a more negative Δ*G* indicates a higher reactivity, the reaction Al + NH_3_ → AlN + 3/2 H_2_ has a higher reactivity than the reaction Al + 1/2N_2_ → AlN. However, it should be noted that Δ*G* for the latter is also negative, indicating that a spontaneous reaction between Al and N_2_ occurs near 1500 °C at 100 kPa. If AlN single crystals can be grown using the reaction Al + 1/2N_2_ → AlN, many benefits should be realized, including an environmentally friendly process without hazardous species (which leads to the exclusion of a detoxifying apparatus), absence of side products, and cost effectiveness.

However, direct nitridation of Al metal is unsuitable for single crystal AlN growth because N_2_ gas supplied onto an Al source at an elevated temperature creates AlN polycrystals on the molten Al surface, and eventually an AlN crust coats the Al source, preventing further AlN growth^32^. To overcome this issue, we designed a vapor-phase-epitaxy-based growth apparatus, as schematically illustrated in Fig. 1b. The horizontal reactor has two temperature zones: one to generate Al vapor and the other for AlN growth. These zones can be operated at different temperatures. Furthermore, the source zone is divided into the upper and lower channels. Inert argon (Ar) gas introduced into the lower channel transfers the heat-generated Al vapor to the growth zone. On the other hand, N_2_ is supplied into the upper channel in order to avoid premature reactions with Al vapor before reaching the substrate. These gases join together in the growth zone, where the substrates are placed. This growth technique enables Al vapor to be supplied continuously without forming an AlN crust, allowing crystal growth to proceed at a given growth rate. Figure 1c shows a photograph of the growth apparatus in operation. (The growth process is described in detail in Methods).

### Confirming the reactivity between Al and N_2_

**Figure 2:**
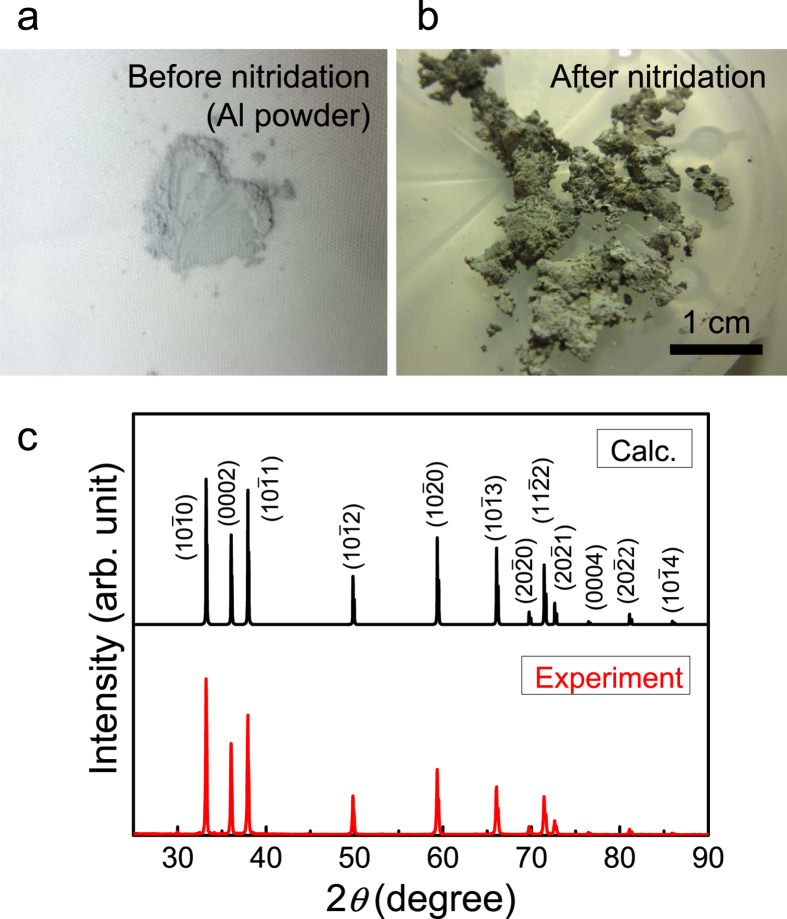
Direct nitridation of the Al source metal by N_2_ gas. (**a,b**) Photographs of the Al source powder before nitridation and the product powder after nitridation, respectively. (**c**) Calculated XRD profile of AlN powder (upper) and experimental XRD profile of the nitrided powder (lower).

Before trying to grow an AlN single-crystal on a substrate, we confirmed the reactivity between Al and N_2_ by directly supplying N_2_ (instead of Ar) gas onto Al powders. Figure 2a,b how photographs of the Al source powder before nitridation and the product powder after nitridation, respectively. Nitridation was carried out at 1300 °C at 95 kPa for 1 hr.

Because nitridation affects the color and grain size, the crystallographic states were investigated with x-ray diffraction (XRD) measurements. Figure 2c shows the simulated profile of the AlN polycrystalline powder (upper) and the experimentally obtained profile after nitridation (lower). The simulation assumed a hexagonal wurtzite structure, which is the most stable structure for AlN. The good agreement between the simulation and experiment indicates that AlN polycrystals are successfully synthesized via direct nitridation.

Composition analyses via energy dispersive x-ray spectroscopy reveal that most of the powder is pure AlN, but some is an AlN shell/Al core composite, which is most likely due to the formation of an AlN crust on the powder (Supplementary information). The optical properties of the synthesized AlN powder are described in the Supplementary information. Concisely, cathodoluminescence from each AlN powder exhibits a broad but intense emission at ~360 nm at RT, suggesting that AlN may possess an alluring feature as a near ultraviolet phosphor. These experimental findings have encouraged us to grow AlN single crystals with our growth apparatus shown in Fig. 1b,c.

### Growth and characterization of thick AlN single crystals from Al and N_2_

**Figure 3:**
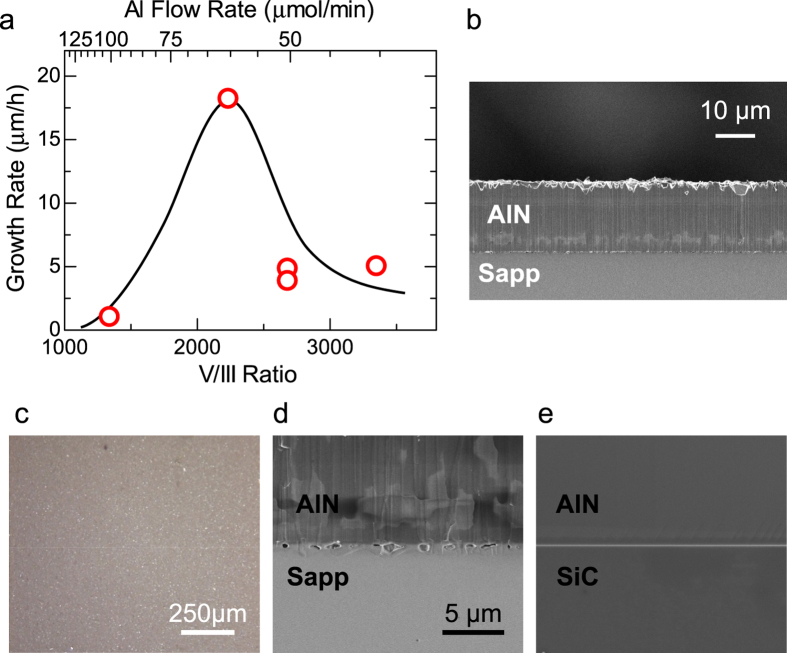
Structural properties of AlN grown from Al and N_2_. (**a**) Growth rate as a function of the V/III ratio at 1550 °C. Maximum growth rate is ~18 μm/h when the V/III ratio is ~2200. (**b**) Cross-sectional SEM image of the 18-μm-thick AlN layer. (**c**) Surface optical microscopy image of the same AlN shown in (**b**) without cracks, which greatly differs from AlN on SiC grown in this study as well as MOVPE AlN on sapphire. (**d,e**) Close-up images of the AlN/sapphire and AlN/SiC interfaces, respectively. Both use the same scale. Voids form only at the AlN/sapphire interface.

To grow AlN, the source raw materials were Al powder (purity = 99.99%) and N_2_ gas, while the substrates were sapphire(0001) unless stated. Typical source zone temperature and growth pressure were 1400 °C and 10 kPa, respectively, which were determined in the manner described in Methods. Growth optimization was performed for the growth zone temperature and the molar flow ratio of N_2_ to Al, which is referred to as the V/III ratio in this letter. The optimized growth zone temperature was 1550 °C (Supplementary information). It is noteworthy that the optimal growth temperature in this study is much lower than that in sublimation (~2000 °C). To adjust the V/III ratio, the Al flow rate was changed under a constant N_2_ flow rate of 3 standard l/min. Figure 3a plots the growth rate at the optimal growth temperature of 1550 °C as a function of the V/III ratio. Very low V/III ratios tend to form AlN whiskers, which slows the film growth rate. This trend is consistent with GaN MOVPE, in which a low V/III ratio reduces the driving force for growth and promotes nanowire formation^33^. Additionally, V/III ratios greater than 2500 decrease the growth rate simply because the Al flow rate that contributes to growth is reduced (see the upper axis of Fig. 3a). Therefore, in terms of the growth rate, the optimal V/III ratio in this study is ~2200.

Figure 3b exhibits a cross-sectional scanning electron microscopy (SEM) image of the thickest AlN layer grown under the optimal growth conditions. The maximum growth rate achieved in this study is ~18 μm/h. This rate is slower than that of sublimation^22^, but is comparable to that of HVPE (~25 μm/h)^20^. Further increasing the Al flow rate is expected to result in a faster growth rate.

Figure 3c shows an optical microscopy image of the same AlN surface. Usually, AlN films thicker than 1 μm on sapphire(0001) involve cracks^34^,^35^ due to the tensile strain in AlN induced by the mismatch in the thermal expansion. However, cracks do not exist in our AlN even though it is 18-μm thick. To better understand this observation, Fig. 3d shows a magnified SEM image at the AlN/sapphire interface where many voids, which measure a few hundred nanometers, are observed. On the other hand, in AlN grown on a co-loaded 6H-SiC(0001) substrate, such interfacial voids do not exist (Fig. 3e). Interestingly, many cracks form in AlN on 6H-SiC. Similarly, MOVPE AlN on sapphire involves cracks but not voids. These findings lead us to conclude that the interfacial voids act as (tensile) strain absorbers, which prevent cracks from being induced.

Because the samples shown in Fig. 3d,e were simultaneously grown, comparing them can help clarify the void formation mechanism. Because voids are absent at the AlN/SiC interface, the reactions between the source precursors and sapphire substrates seem to be responsible for void formation at the AlN/sapphire interface. We presume that the Al vapor can etch sapphire (Al_2_O_3_) because Al, which has a strong reducing ability, may cause the reaction Al_2_O_3_ + 4Al → 3Al_2_O, where Al_2_O is in the gas phase at the growth temperature.

**Figure 4:**
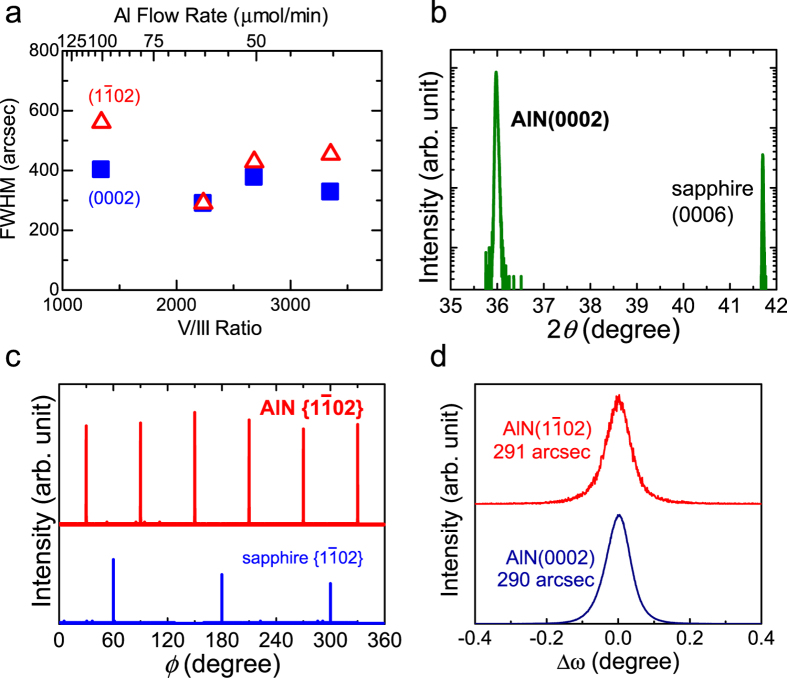
Crystallographic properties of 18-μm-thick AlN assessed by XRD. (**a**) Plot of FWHMs of the *ω*-scan of the AlN symmetric (0002) and the asymmetric (1
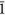
02) planes as functions of the V/III ratio. (**b**) 2*θ*/*ω* profile of the symmetric plane. Single peak of AlN(0002) indicates [0001]-oriented growth of wurtzite AlN. (**c**) *ϕ*-scans of the asymmetric AlN and sapphire {1
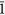
02} planes. Six-fold symmetry of AlN indicates that AlN is a single-phase crystal. Disagreement between the AlN and sapphire peaks indicate an in-plane rotation by 30° to minimize the lattice mismatch. (**d**) *ω*-scan of the AlN symmetric (0002) and the asymmetric (1
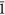
02) planes. Line widths at the half maximum are as narrow as 290 and 291 arcsec, respectively.

The crystallinity of the grown AlN layers was assessed by XRD measurements. Figure 4a shows the full widths at half maximum (FWHMs) of the *ω*-scan of the AlN symmetric (0002) and asymmetric (1
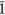
02) diffractions as functions of the V/III ratio during growth. Although the variation due to the V/III ratio is insignificant, the narrowest widths are obtained with V/III~2200. In addition, V/III~2200 provides the fastest growth rate in this study (Fig. 3a), confirming that it is the best growth condition. Therefore, the results for the 18-μm-thick AlN layer on sapphire(0001) grown with V/III~2200 are used below as a representative material. Figure 4b shows the symmetric 2*θ*/*ω* profile. Apart from sapphire(0006), only the AlN(0002) diffraction is detected, indicating that the AlN layer is a [0001]-oriented wurtzite crystal. The crystallographic orientation in the (0001) plane is examined with *ϕ* scans of the AlN and sapphire asymmetric {1
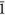
02} planes (Fig. 4c). The clear six-fold symmetry observed for AlN indicates that AlN has a single-phase hexagonal wurtzite structure exclusive of other rotation domains. The angular difference between the AlN and sapphire diffractions is 30° due to the in-plane 30° rotation to mitigate the lattice mismatch, which is often reported for MOVPE or MBE nitride semiconductors on sapphire(0001)^30^,^36^. (The three-fold symmetry of sapphire originates from the crystal structure of trigonal corundum.)

Figure 4d shows the *ω*-scan profiles of the AlN symmetric (0002) and asymmetric (1
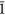
02) diffractions, which have FWHMs of 290 and 291 arcsec, respectively. From these values, the screw and edge dislocation densities (*N*) can be estimated using the equation *N* = FWHM^2^/4.35|***b***|^2^, where ***b*** is the Burgers vector of the corresponding dislocations^37^. For screw dislocations, ***b*** = [0001] and FWHM of the tilt component [*i.e.,* (0002) FWHM] must be used. For edge dislocations, ***b*** = 1/3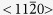 and FWHM of the twist component, which is determined by (FWH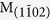)^2^ = (FWHM_tilt_ cos*χ*)^2^ + (FWHM_twist_ sin*χ*)^2^, must be used. Here *χ* is the angle between the (0001) and (1
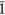
02) planes. The estimated screw and edge dislocation densities are 1.8 × 10^8^ and 4.7 × 10^8^ /cm^2^, respectively. It is noteworthy that the total dislocation density is one of the lowest among the reported values for AlN-on-sapphire or AlN-on-SiC heterostructures, demonstrating the superiority of our proposed method. (A detailed summary is given in Supplementary information).

**Figure 5:**
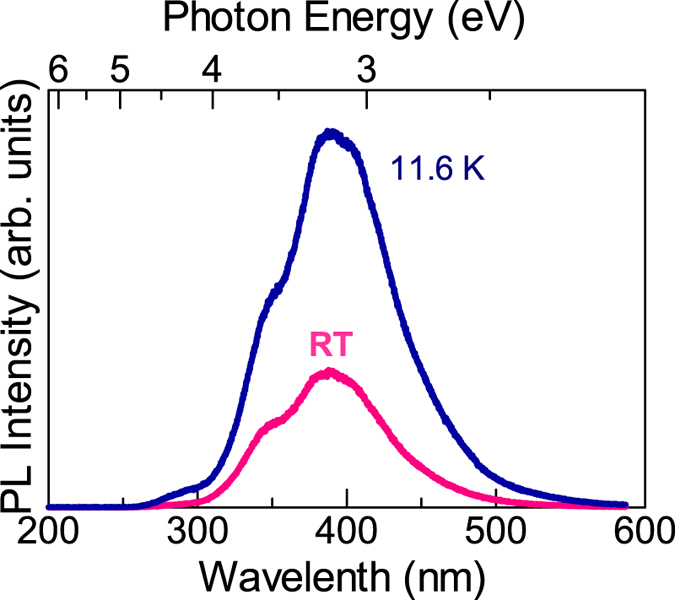
Optical properties of 18-μm-thick AlN assessed by PL at 11.6 K and RT. Because the emission is located at ~390 nm, it is not band edge emission, but a deep level emission that is likely related to Al vacancies and deep donors. Intensity ratio between RT and 11.6 K suggests a high internal quantum efficiency of ~32%.

The optical properties were assessed by photoluminescence (PL). (See Methods.) Figure 5 shows the PL spectra of the 18-μm-thick AlN layer acquired at RT and 11.6 K. The band edge emission near 6 eV is absent, but a deep level emission is observed at ~390 nm (~3.2 eV). The ratio of the PL integrated intensity at RT against that at 11.6 K is as high as 32%, which is an approximation of the internal quantum efficiency at RT if nonradiative recombination processes at low temperatures are negligible.

Furthermore, the shoulders at ~340 nm (~3.7 eV) and ~290 nm (~4.3 eV) indicate that several different origins are superimposed. To date, the deep level emissions in AlN have been discussed in terms of the Al-vacancies (*V*_Al_) and impurities such as oxygen donors occupying the nitrogen site in AlN (O_N_)^38^,^39^. Generally, cation vacancies in compound semiconductors form deep acceptor levels because they leave electron-deficient states that accept electrons in a material. Such deep acceptor levels have been reported not only for nitride semiconductors^40^,^41^,^42^, but also for many other semiconductors, including GaAs^43^, ZnSe^44^, and ZnGeP_2_^45^.

The Al-vacancies in AlN are triple acceptors (*V*_Al_)^3−^, and vacancy-impurity complexes such as (*V*_Al_ - O_N_)^2−^ and (*V*_Al_ - 2O_N_)^1−^ also form deep acceptor levels^38^,^39^,^41^. The transitions between these three deep acceptor levels and the donor level appear around 3.4, 3.9, and 4.7 eV, respectively^38^. The reported emission energies agree fairly well with the experimentally observed PL components, suggesting the relevance of such transitions in our AlN.

To confirm the origin of luminescence, we fabricated another AlN sample under an extreme Al excess (low V/III ratio), which was realized by directly soaking a sapphire substrate into the Al source and supplying N_2_ on it. As described above and in the Supplementary information, such a low V/III ratio creates AlN whiskers. A new emission band emerges around 6 eV (~210 nm) in the cathodoluminescence of the fabricated AlN whiskers, suggesting that the excess Al source suppresses *V*_Al_ formation and some carriers recombine radiatively at the band edge without being captured by *V*_Al_, which is responsible for the deep level emission.

Then we estimated the oxygen concentration in an AlN thick layer by secondary ion mass spectroscopy. (See Methods.) The concentration is 3 × 10^18^ /cm^3^, indicating the presence of oxygen in our AlN. It is noteworthy that our AlN layer is nitrogen polar (see Supplementary information), which may enhance impurity incorporation, as often reported for GaN^46^,^47^.

Although vacancies and impurities should be reduced in the future, safe source materials, moderate growth temperature, relatively high growth rate, and superior crystalline quality of AlN grown using the proposed method strongly indicate that the proposed method is a significant step toward environmentally friendly crystal growth processes. The proposed growth method is named as Elementary source Vapor Phase Epitaxy (EVPE), and is applicable to homoepitaxy in future studies to further improve quality.

## Methods

### AlN growth

The proposed growth is a type of hot-wall vapor phase epitaxy using two radio-frequency induction heating systems. The graphite susceptor allows the temperatures of both the source and growth zones to reach up to 2200 °C. Each temperature is separately monitored and controlled by a thermometer. Although this preliminary study employs Al powder as the starting material, replacing it with Al in a more bulky form may improve the purity.

The flow rates of Ar and N_2_ gases were controlled by mass flow controllers. The Al vapor pressure (*P*/Torr) at a certain temperature (*T*/K) between 1200 and 2800 K is given by log *P* = −16.45×10^3^/*T* + 12.36 − 1.023log*T* ref. 48, which well approximates *P* directly derived from the Gibbs free energy change of the reaction Al(l) → Al(g). Therefore, an Ar flow rate of 1 standard l/min corresponds to an Al flow rate of 100 μmol/min at 1400 °C at 10 kPa, which is comparable to the Al flow rate in MOVPE. That is, adjusting the Ar flow rates under these conditions results in reasonable Al flow rates. Additionally, because a lower pressure tends to decrease Δ*G* (ref. 49) and promotes the reaction Al + 1/2N_2_ → AlN, this study reduced the growth pressure to 10 kPa.

### PL measurements

The excitation source was a pulsed ArF excimer laser (λ = 193 nm, τ = 4 ns, and 25 mJ/cm^2^/pulse). The laser beam was focused onto a spot with a 100-μm diameter. The spectra were acquired by a 50-cm monochromator (resolution: 0.4 nm) in conjunction with a liquid-N_2_-cooled CCD camera.

### Secondary ion mass spectroscopy (SIMS)

To evaluate the oxygen concentration in our AlN, SIMS measurements were performed using Cs^+^ as an incident ion with an acceleration voltage of 15.0 kV with a 30-μm-square measurement area. The oxygen concentration was quantified by a standard AlN sample established prior to the measurement.

## Additional Information

**How to cite this article**: Wu, P.T. *et al.* Environmentally friendly method to grow wide-bandgap semiconductor aluminum nitride crystals: Elementary source vapor phase epitaxy. *Sci. Rep.* **5**, 17405; doi: 10.1038/srep17405 (2015).

## Supplementary Material

Supplementary Information
