# Supplementary material for: Environmentally friendly method to grow wide-bandgap semiconductor aluminum nitride crystals: Elementary source vapor phase epitaxy
Source: Sci Rep. 2015 Nov 30;5:17405. doi: 10.1038/srep17405 (PMC4663762; doi:10.1038/srep17405)
Supplement: Supplementary Information [file srep17405-s1.pdf]

Supplementary Information

Environmentally friendly method to grow wide-bandgap semiconductor aluminum nitride crystals: Elementary source vapor phase epitaxy

PeiTsen Wu, Mitsuru Funato, and Yoichi Kawakami

Department of Electronic Science and Engineering, Kyoto University, Kyoto 615-8510, Japan

Supplementary Information S1

Characterizations of AlN powders synthesized by direct nitridation of Al

Direct nitridation of Al powders creates AlN polycrystalline powders (Fig. 2). Figure S1a shows a scanning electron microscopy (SEM) image of AlN powder, which has diameters ranging from less than 1 μm to more than 5 μm. Figure S1b shows the average cathodoluminescence (CL) spectrum of the area shown in Fig. S1a acquired at room temperature (RT) with an acceleration voltage of 10 kV and an electron beam current of 0.43 nA. The emission peak is located at ~360 nm, which is not a band edge emission but a deep level emission probably related to the Al vacancies and deep donor complexes, similar to the photoluminescence (PL) of the AlN layer in Fig. 5. Figure S1c shows the CL map acquired at RT at the same position as the SEM image shown in Fig. S1a with a monitoring wavelength near the CL peak (360 nm). Many AlN powders exhibit clear CL.

Figure S1d shows the spectra of the energy dispersive x-ray spectroscopy (EDS) acquired at positions 1 and 2 designated in Fig. S1a. The irradiated electron beam conditions are the same as those in the CL measurements. Generally, the N signal is much weaker than the Al signal, mostly due to the difference in the x-ray generation yields of Al and N atoms and partly due to the sensitivity of the measurement system. To correct for these fatal factors, an AlN thick film grown by metalorganic vapor phase epitaxy (MOVPE) was also measured as a standard. The AlN powder at position 1 is pure AlN (despite the largely different Al and N EDS signal intensities), whereas that at position 2 is Al-rich. The latter may be attributed to the formation of an AlN crust around an Al powder, as reported in ref. S1.

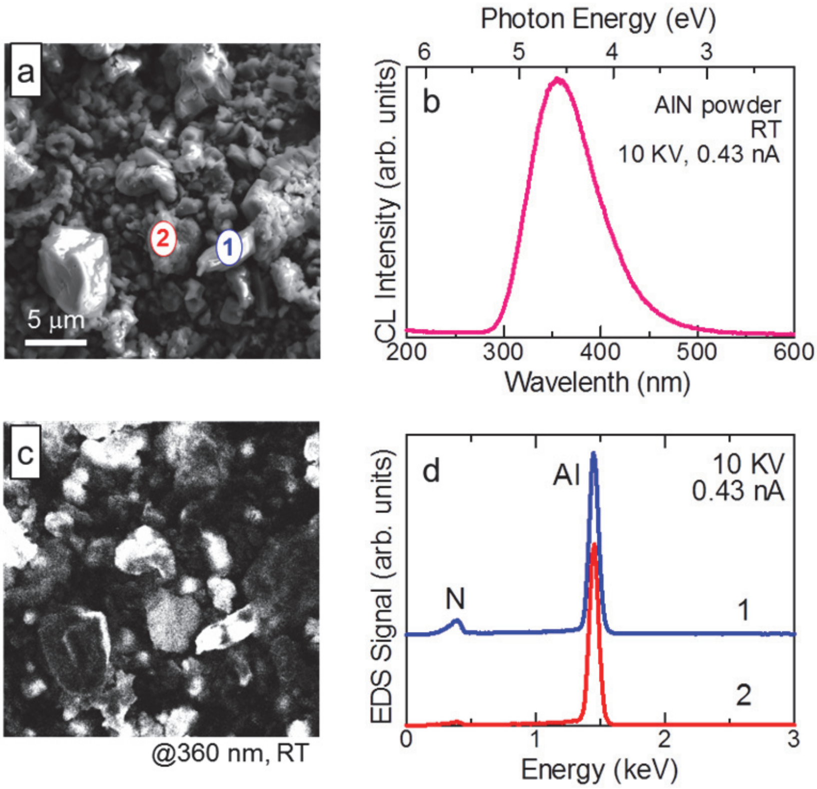

Figure S1 **Characterization of AlN powders synthesized via a direct reaction between Al and N_2.** **a** SEM image. **b** CL spectrum acquired at RT in the area shown in the SEM image **a**. Emission peak at ~360 nm is related to deep levels. **c** CL mapping of the same area as the SEM image in **a** monitored at 360 nm indicates emissive AlN powders. **d** EDS signals taken at positions 1 and 2 designated in **a**. At position 1, the EDS signal indicates nearly stoichiometric AlN, whereas at position 2, the formation of an AlN shell / Al core structure decreases the N signal.

To quantify the thickness of the AlN crust, a Monte Carlo scheme was used to simulate the trajectory of the irradiated electron. The assumed structure was an AlN flat layer with a variable thickness on infinitely thick Al. The experimentally observed Al and N EDS signal intensity ratios at position 2 are reproduced using 70-nm-thick AlN on Al. Thus, most of the powder is composed of AlN, but some consists of the AlN shell / Al core structure. It is noteworthy that an intense CL is obtained even from the composite structure as

shown in Fig. S1c, suggesting that the AlN shell has a high optical quality.

Macroscopic PL was measured at 13 K and RT under the same conditions as Fig. 5. Assuming negligible non-radiative processes and an internal quantum efficiency (IQE) of 100% at low temperatures, the PL intensity ratio provides the IQE at a measured temperature; the IQE of our AlN powder at RT is as high as 59%, which greatly exceeds that of the AlN layer shown in Fig. 5 (32%). The higher IQE is most likely due to a smaller dislocation density in the powder. This along with the results shown in Fig. S1 suggests that the AlN powder is promising as an UV phosphor.

Reference

[S1] Zhang, D., Liu, F.M., Cai, L.G., Liu, X.Q. & Li. Y. Formation of novel core-shell and tadpole-like structures in the direct nitridation of aluminum powder by N_2 and NH_3. *J. Alloys. Comp.* **547**, 91-99 (2013).

Supplementary Information S2

Growth temperature optimization for AlN thick layers

AlN layers were grown from Al and N_2 on sapphire(0001) at different temperatures at a constant V/III ratio of 2700. Figure S2 shows the variation in the growth morphology as a function of temperature. Increasing the temperature from 1500 to 1550°C increases the film thickness, while the XRD measurements indicate that the film remains in a single-phase AlN. However, increasing the temperature to 1600°C causes AlN whisker to form, which may be due to the decomposition of the sapphire substrate. Hence, the growth conditions at 1550°C were optimized, as described in the main text.

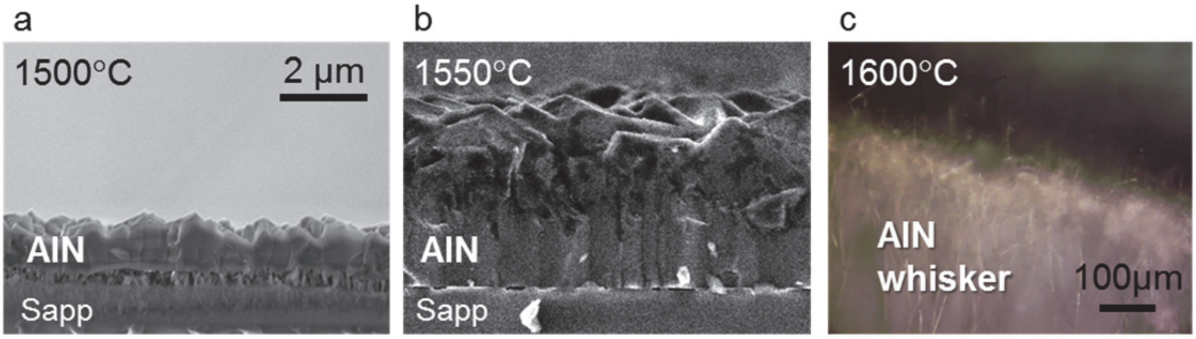

Figure S2 Growth morphology of AlN as a function of temperature. **a**, **b** Cross-sectional SEM images (the scale bar is common) and **c** top view observed with an optical microscope.

Supplementary Information S3

Crystalline quality of AlN grown on foreign substrates

We investigated the full widths at half maximum (FWHMs) of the x-ray diffraction (XRD) patterns of AlN grown on foreign substrates (sapphire and SiC) by hydride vapor phase epitaxy (HVPE)[S2-S30] or sublimation[S31-S36] reported in the last decade. Figure S3 plots the results of both symmetric (0002) and asymmetric ($1\bar{1}02$) FWHMs. [If another asymmetric plane is used, the FWHM for ($1\bar{1}02$) is estimated.] Note that the AlN thickness is on the order of a few tens μm for HVPE, but is a few mm for sublimation, which may cause the difference in FWHMs. The symmetric (0002) diffraction reflects the tilt

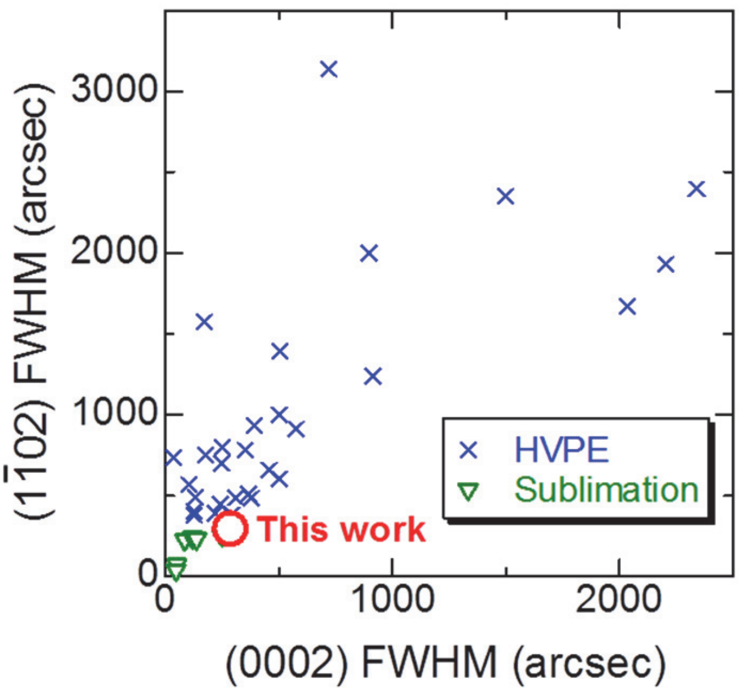

Figure S3 Summary of the XRD FWHMs of AlN grown by HVPE or sublimation. Symmetric (0002) diffraction reflects the tilt component, whereas asymmetric ($1\bar{1}02$) diffraction includes the contribution from the twist component. Open circle indicates the result of this study.

component determined by screw dislocations, while the asymmetric $(1\bar{1}02)$ diffraction includes the contribution from the twist component determined by edge dislocations. Therefore, both FWHMs should be narrow. Our sample has structural properties superior to HVPE AlN with a similar thickness (a few tens μm). On the other hand, AlN grown by sublimation has narrower line widths, which may be due to the aforementioned larger crystal dimension because as growth proceeds, dislocations tend to be terminated (e.g., by dislocation pairing). In other words, increasing the AlN layer thickness with our method should lead to FWHMs similar to those by sublimation.

Reference

[S2] Gong, X. *et al*. Evolution of the surface morphology of AlN epitaxial film by HVPE. *J. Cryst. Growth* **409**, 100-104 (2015).

[S3] Claudel, A. *et al*. Influence of the V/III ratio in the gas phase on thin epitaxial AlN layers grown on (0001) sapphire by high temperature hydride vapor phase epitaxy. *Thin Solid Films* **573**, 140-147 (2014).

[S4] Baker, T., Mayo, A., Veisi, Z., Lu, P. & Schmitt, J. Hydride vapor phase epitaxy of AlN using a high temperature hot-wall reactor. *J. Cryst. Growth* **403**, 29-31 (2014).

[S5] Kitagawa, S., Miyake, H. & Hiramatsu, K. High-quality AlN growth on 6H-SiC substrate using three dimensional nucleation by low-pressure hydride vapor phase epitaxy. *Jpn. J. Appl. Phys.* **53**,

05FL03 (2014).

[S6] Togashi, R. *et al*. Influence of source gas supply sequence on hydride vapor phase epitaxy of AlN on (0001) sapphire substrates. *J. Cryst. Growth* **360**, 197-200 (2012).

[S7] Okumura, K., Nomura, T., Miyake, H., Hiramatsu, K. & Eryuu, O. HVPE growth of AlN on trench-patterned 6H-SiC substrates. *Phys. Status Solidi c* **8**, 467-469 (2011).

[S8] Bryant, B. N., Kamber, D. S., Wu, F., Nakamura, S. & Speck, J. S. Aluminum nitride grown on lens shaped patterned sapphire by hydride vapor phase epitaxy. *Phys. Status Solidi c* **8**, 1463-1466 (2011).

[S9] Fujita, K., Okuura, K., Miyake, H., Hiramatsu, K. & Hirayama, H. HVPE growth of thick AlN on trench-patterned substrate. *Phys. Status Solidi c* **8**, 1483-1486 (2011).

[S10] Volkova, A., Ivantsov, V. & Leung, L. Hydride vapor phase epitaxy of high structural perfection thick AlN layers on off-axis 6H-SiC. *J. Cryst. Growth* **314**, 113-118 (2010).

[S11] Kumagai, Y. *et al*. Investigation of void formation beneath thin AlN layers by decomposition of sapphire substrates for self-separation of thick AlN layers grown by HVPE. *J. Cryst. Growth* **312**, 2530-2536 (2010).

[S12] Wu, J. J., Okuura, K., Miyake, H. & Hiramatsu, K. Effects of substrate plane on the growth of high quality AlN by hydride vapor phase epitaxy. *Appl. Phys. Exp.* **2**, 111004 (2009).

[S13] Katagiri, Y., Kishino, S., Okuura, K., Miyake, H. & Hiramatu, K. Low-pressure HVPE growth of crack-free thick AlN on a trench-patterned AlN template. *J. Cryst. Growth* **311**, 2831-2833

(2009).

[S14] Tajima, J., Murakami, H., Kumagai, Y., Takada, K. & Koukitu, A. Preparation of a crack-free AlN template layer on sapphire substrate by hydride vapor-phase epitaxy at 1450°C. *J. Cryst. Growth* 311, 2837-2839 (2009).

[S15] Soukhoveev, V. *et al*. Large area GaN and AlN template substrates fabricated by HVPE. *Phys. Status Solidi c* **6**, S333-S335 (2009).

[S16] Eriguchi, K., Hiratsuka, T., Murakami, H., Kumagai, Y. & Koukitu, A. High-temperature growth of thick AlN layers on sapphire (0001) substrates by solid source halide vapor-phase epitaxy. *J. Cryst. Growth* **310**, 4016-4019 (2008).

[S17] Kumagai, Y. *et al*. Self-separation of a thick AlN layer from a sapphire substrate via interfacial voids formed by the decomposition of sapphire. *Appl. Phys. Exp.* **1**, 045003 (2008).

[S18] Tajima, J. *et al*. Growth of thin protective AlN layers on sapphire substrates at 1065 degrees C for hydride vapor phase epitaxy of AlN above 1300°C. Phys. Status Solidi c 5, 1515-1517 (2008).

[S19] Nagashima, T. *et al*. Improvement of AlN crystalline quality with high epitaxial growth rates by hydride vapor phase epitaxy. *J. Cryst. Growth* **305**, 355-359 (2007).

[S20] Tsujisawa, K. *et al*. Suppression of crack generation using high-compressive-strain AlN/Sapphire template for hydride vapor phase epitaxy of thick AlN film. *Jpn. J. Appl. Phys.* **46**, L552-L555 (2007).

[S21] Nagashima, T. *et al*. High-speed epitaxial growth of AlN above 1200°C by hydride vapor

phase epitaxy. *J. Cryst. Growth* **300**, 42-44 (2007).

[S22] Eriguchi, K. *et al*. MOVPE-like HVPE of AlN using solid aluminum trichloride source. *J. Cryst. Growth* **298**, 332-335 (2007).

[S23] Tsujisawa, K. *et al*. High temperature growth of AlN film by LP-HVPE. *Phys. Status Solidi c* **4**, 2252-2255 (2007).

[S24] Sukhoveev, V. *et al*. Thick AIN layers grown by HVPE on sapphire substrates. *MRS Proc.* **892**, 743-748 (2006).

[S25] Liu, Y. H. *et al*. Fabrication of thick AlN film by low pressure hydride vapor phase epitaxy. *Phys. Status Solidi c* **3**, 1479-1482 (2006).

[S26] Soukhoveev, V. *et al*. Recent results on AIN growth by HVPE and fabrication of free standing AlN wafers. *Phys. Status Solidi c* **3**, 1653-1657 (2006).

[S27] Kumagai, Y., Yamane, T. & Koukitu, A. Growth of thick AlN layers by hydride vapor-phase epitaxy. *J. Cryst. Growth* **281**, 62-67 (2005).

[S28] Kovalenkov, O., Soukhoveev, V., Ivantsov, V., Usikov, A. & Dmitriev, V. Thick AlN layers grown by HVPE. *J. Cryst. Growth* **281**, 87-92 (2005).

[S29] Yamane, T., Murakami, H., Kangawa, Y., Kumagai, Y. & Koukitu, A. Growth of thick AlN layer on sapphire (0001) substrate using hydride vapor phase epitaxy. *Phys. Status Solidi c* **2**, 2062-2065 (2005).

[S30] Bliss, D. F., Tassev, V. L., Weyburne, D. & Bailey, J. S. Aluminum nitride substrate growth by

halide vapor transport epitaxy. *J. Cryst. Growth* **250**, 1-6 (2003).

[S31] Sumathi, R. R. & Gille, P. Role of SiC substrate polarity on the growth and properties of bulk AlN single crystals. *J. Mater Sci: Mater. in Elec.* **25**, 3733-3741 (2014).

[S32] Sumathi, R. R. & Gille, P. Development and progress in bulk c-plane AlN single-crystalline template growth for large-area native seeds. *Jpn. J. Appl. Phys.* **52**, 08JA02 (2013).

[S33] Hartmann, C. *et al*. SiC seed polarity-dependent bulk AlN growth under the influence of residual oxygen. *J. Cryst. Growth* **344**, 19-26 (2012).

[S34] Bickermann, M. *et al*. Growth of AlN bulk crystals on SiC seeds: Chemical analysis and crystal properties. *J. Cryst. Growth* **339**, 13-21 (2012).

[S35] Yamakawa, M. *et al*. Freestanding highly crystalline single crystal AlN substrates grown by a novel closed sublimation method. *Appl. Phys. Exp.* **4**, 045503 (2011).

[S36] Miyanaga, M. *et al*. Evaluation of AlN single-crystal grown by sublimation method. *J. Cryst. Growth* **300**, 45-49 (2007).

Supplementary Information S4

AlN whiskers formed under an Al excess

To confirm the presence of Al-vacancies in our AlN, AlN was grown under a large Al excess (low V/III ratio) by directly soaking a sapphire substrate into the Al source and supplying N_2 on it. Such conditions form AlN whiskers. Figures S4a and b show the panchromatic CL mapping image and spectra of the generated AlN whiskers at RT, respectively. The AlN whiskers exhibit a near-band-edge excitonic emission around 6 eV (~210nm), which indicates that the excess Al successfully reduces the number of Al-vacancies and the excited carriers can recombine at the band edge without being captured by the deep levels responsible for the emission at ~360 nm.

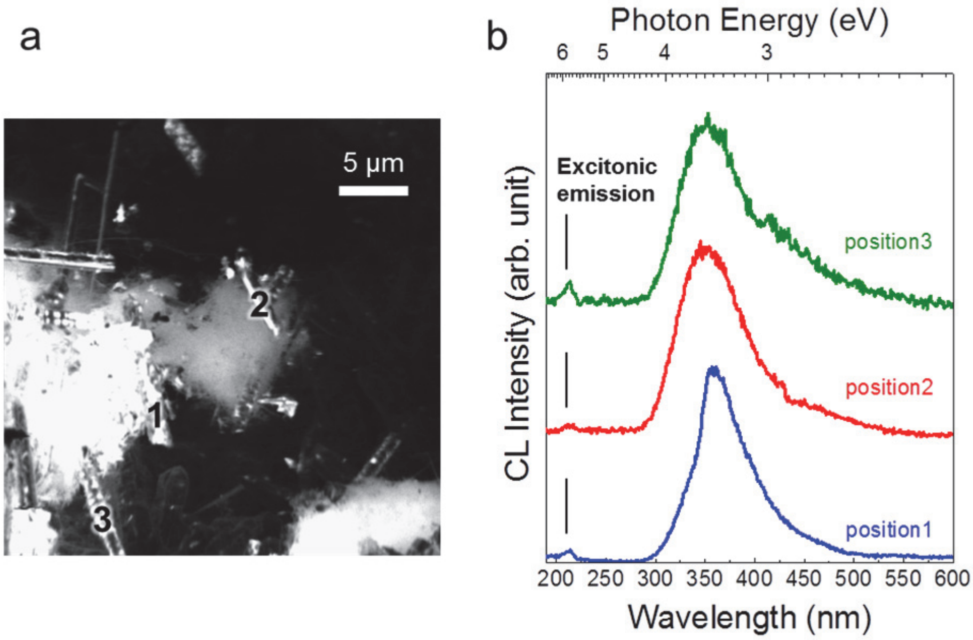

Figure S4 CL properties of AlN grown under excess Al. **a** Panchromatic CL map and **b** CL spectra acquired at positions designated in **a**. All measurements are at RT.

Supplementary Information S5

Polarity of AlN grown in this study

To determine the polarity of the grown AlN layers, the convergent-beam electron diffraction (CBED) patterns were acquired along the AlN $[11\bar{2}0]$ direction. For the CBED measurements, specimens were prepared by the conventional Ar^+ milling technique. The acceleration voltage for the electron beam was 200 kV.

Figure S5 shows the CBED pattern. A marked contrast due to the difference in Al and N should appear in the (0002) diffraction disk,[S37] suggesting that our AlN layers are N-polar. This assertion is further supported by a simulation reported in ref. S37 where the simulated CBED pattern for a 100-nm-thick specimen with an electron acceleration voltage of 200 kV resembles the pattern shown in Fig. S5.

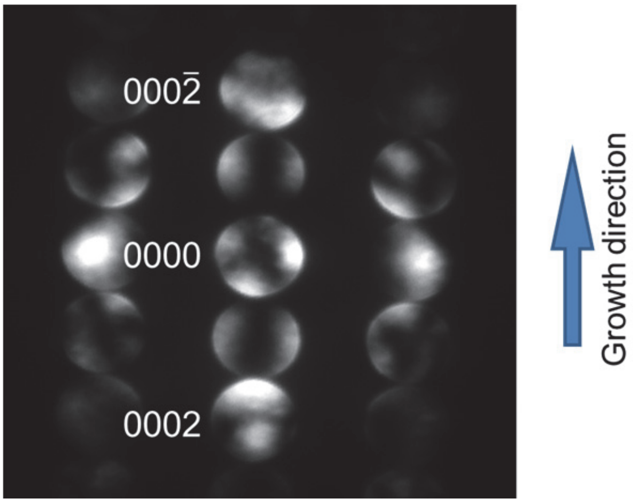

Figure S5 CBED pattern of the AlN layer grown in this study. Electron beam is incident along the $[11\bar{2}0]$ direction. (0002) and $(000\bar{2})$ disks differ.

AlN films grown on sapphire(0001) by MOVPE are usually Al-polar. These findings suggest that the crystal growth mechanism of AlN prepared in this work differs from that grown by MOVPE. Therefore, the crystal growth mechanism in the proposed method should be further investigated.

Reference

[S37] Imura, M. *et al*. Analysis of broken symmetry in convergent-beam electron diffraction along <$11\bar{2}0$> and <$1\bar{1}00$> zone-axes of AlN for polarity determination. *Jpn. J. Appl. Phys.* **52**, 08JE15 (2013).
